# Physiotherapy Combined With Voice Exercises in a Patient With Unilateral Vocal Cord Palsy Following a Total Thyroidectomy Surgery: A Case Report

**DOI:** 10.7759/cureus.35217

**Published:** 2023-02-20

**Authors:** Fatimah Kazi, Shubhangi Patil, Heena Pathan

**Affiliations:** 1 Community Health Physiotherapy, Ravi Nair Physiotherapy College, Datta Meghe Institute of Higher Education and Research, Wardha, IND

**Keywords:** voice exercises, rehabilitation, total thyroidectomy, physiotherapy, voice therapy, unilateral vocal cord palsy

## Abstract

Multinodular goiter is a condition in which the thyroid gland is swollen and has several distinct masses. A large multinodular goiter can lead to difficulty in swallowing and breathing. A large goiter hampers respiration and deglutition; therefore, a part of or the whole thyroid gland is removed. Total thyroidectomy is a surgical process which involves the removal of the whole thyroid gland. One of the adverse effects of a complete thyroidectomy is vocal cord paralysis. It occurs because of an injury to the recurrent laryngeal nerve. Vocal cord paralysis could be bilateral or unilateral. It is characterized by hoarseness of voice, breathing difficulties and voice pitch loss, and inability to talk loudly. This case report describes physiotherapy along with voice exercises in a 65-year-old female who suffered from unilateral vocal cord palsy following total thyroidectomy. The patient was successfully rehabilitated after four weeks, using a tailored physiotherapy program according to the difficulty faced by her. The rehabilitation exercises consisted of upper and lower limb mobility activities, breathing activities including thoracic expansion, and deep breathing exercises. Static hamstrings, static quadriceps exercise, heel slides and isometric exercise to neck muscles, and passive movements to the cervical spine were administered. Voice therapy exercises combined with breathing exercises were also administered.

## Introduction

An enlarged thyroid gland is known as goiter. It can occur due to hypothyroidism (a condition in which the thyroid gland does not produce a sufficient quantity of thyroid hormone) and hyperthyroidism (a condition in which an excessive amount of thyroid hormone is made). The incidence of multinodular goiter is dependent on iodine intake. Multinodular goiter is when the thyroid gland is swollen and has several distinct nodules. People who suffer from this condition have a prominent swelling on the neck. Often, people discover a swelling detected as goiter during a medical checkup. A large multinodular goiter can lead to difficulty in swallowing and breathing. The incidence is high in areas where iodine deficiency prevails, about 0.4-7%. In areas where iodine deficiency is very low, the incidence is low [1]. Five hundred to 600 million people worldwide suffer from multinodular goiter [2].

It is generally diagnosed with biochemical testing such as thyroid-stimulating hormone (TSH) measurements, T3 (triiodothyronine), T4 (thyroxine) levels, and thyroid ultrasonography. New methods like ultrasound-elastography have been developed recently [3]. If there is an aberrant TSH reading, thyroid scintigraphy may also be utilized. Additionally, several cross-sectional imaging techniques, such as positron emission tomography, computed tomography, and magnetic resonance imaging, might be employed, along with fine-needle aspiration biopsy [4]. The risk factors for developing goiter are female sex, iodine deficiency, age above 40 years, menopause, and pregnancy [5].

The treatment of goiter consists of administering radioactive iodine or prescription drugs like methimazole (tapazole) and propylthiouracil in cases of hyperthyroidism. However, a portion of the entire thyroid gland is removed when the goiter grows to enormous proportions and interferes with breathing and deglutition. Total thyroidectomy is the surgical procedure in which the whole thyroid is removed. In some cases, the majority of the thyroid gland is removed, leaving around 2 g of the gland's posterior region on both sides; this is known as a subtotal thyroidectomy. Total thyroidectomy is the preferable procedure for multinodular goiter, Grave’s disease, and thyroid cancer [6]. For years, subtotal thyroidectomy was the surgery of choice. Still, it was found out that it, too, carried the risk of long-term complications and hypothyroidism as much as there is a risk following a total thyroidectomy operation [7]. Total thyroidectomy eradicates the disease's progression, reduces the likelihood of tumor recurrence, and forgoes the significant hazards of undergoing another operative procedure.

Unilateral vocal cord paralysis is a complication of total thyroidectomy [8]. In 0.3-3% of thyroid operations, the recurrent laryngeal nerve is permanently injured, and in 3-8% of procedures, there is temporary paralysis [9]. During a thyroidectomy operation, injury to the recurrent laryngeal nerve leads to vocal cord paralysis [10]. Vocal cord paralysis is characterized by hoarseness of voice, breathing difficulties, pitch loss, and inability to talk loudly. Post-surgical physiotherapy focuses on increasing the lung capacities of the patient, as the respiratory centers become depressed after administration of general anesthesia, and the long-term effect after surgery causes an overall weakness of the respiratory system and weakness of the incised muscles. Exercises combining diaphragmatic breathing (one of the commonly used breathing exercises) and voice therapy exercises help improve unilateral vocal cord palsy-induced difficulty in vocal resonance [11,12]. It also reduces the energy needed for phonation and relieves vocal fatigue. The use of breathing and phonetic methods to improve voice quality as a rehabilitation method is described in this case report. The case report presents a 65-year-old lady who had undergone total thyroidectomy and suffered unilateral vocal cord paralysis. Her condition was managed with the help of medications. Physiotherapy was employed early to improve the patient’s quality of life, reduce suture site pain, increase her range of motion, and help her recuperate to a near-normal state. Treatment of vocal cord palsy patients using physical therapy is a relatively uncharted field from a physiotherapy standpoint.

## Case presentation

A 65-year-old female came with the chief complaint of swelling on the anterior aspect of the neck in the midline. It extended up to the right sternocleidomastoid muscle. The swelling moved with deglutition. The patient did not have associated pain or change in voice. The patient was apparently alright four years back when she noticed a pea-sized swelling which gradually increased to its present size of 8*10 cm. The swelling was associated with dysphagia. The patient does not have significant family history or drug history, which might have led to the development of this condition. With the history as mentioned above, she visited our hospital, and it was advised that she should undergo the following investigation: Ultrasonography (USG) of the neck, USG-guided fine needle aspiration, high-resolution computed tomography (HRCT) thorax, and contrast-enhanced computed tomography (CECT) neck. On investigations, multinodular goiter with right vocal cord palsy was detected. A total thyroidectomy operation was done on 7/11/2022 under general anesthesia. After surgery, the patient was sent to the surgical-intensive care unit (ICU), where she received supportive care in the form of antibiotics, analgesics, synthetic thyroid hormone drugs, and other supportive treatments. Once her hemodynamic conditions were stable, she was moved to the surgery ward. Physiotherapy was started on the fourth postoperative day. The timeline of these events is mentioned in Table 1.

**Table 1:** Timeline of events

Date of Admission	3/11/22
Date of Total thyroidectomy	7/11/22
Date of Physiotherapy referral	11/11/22
Date of Discharge	26/12/22

Clinical findings

The physiotherapist examined the patient in the surgery ward on the fourth postoperative day. She was observed sitting straight. Upon evaluation following the surgery, the patient had a kyphotic and forward head posture, and her lumbar spine was flexed (10°). A bandage was present on the suture site at the initial observation. The patient had hoarseness of voice, very low-pitched sounds, and difficulty speaking. The numerical pain rating scale (NPRS) was employed to quantify pain. The suture site's pain was rated seven out of ten for severity. The cervical range of motion was reduced; the cervical ranges are described in Table 2. 

**Table 2:** Cervical range of motion

Cervical Flexion	0°- 60°
Cervical extension	0°-50°
Rotation: Right	0°-70°
Left	0°-70°
Lateral flexion: Right	0°-35°
Left	0°-35°

Clinical diagnosis

**Figure 1:**
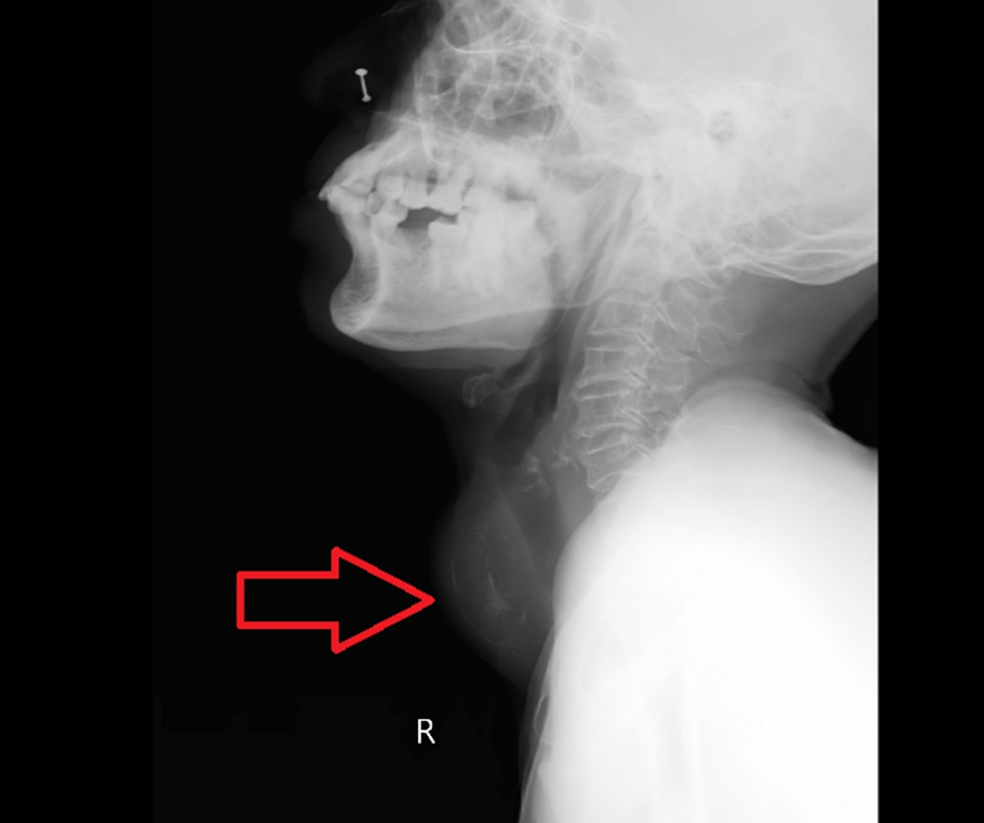
Figure: X-ray of the neck (lateral side) Red arrow: Indicates a globular swelling in-front of the neck

**Figure 2:**
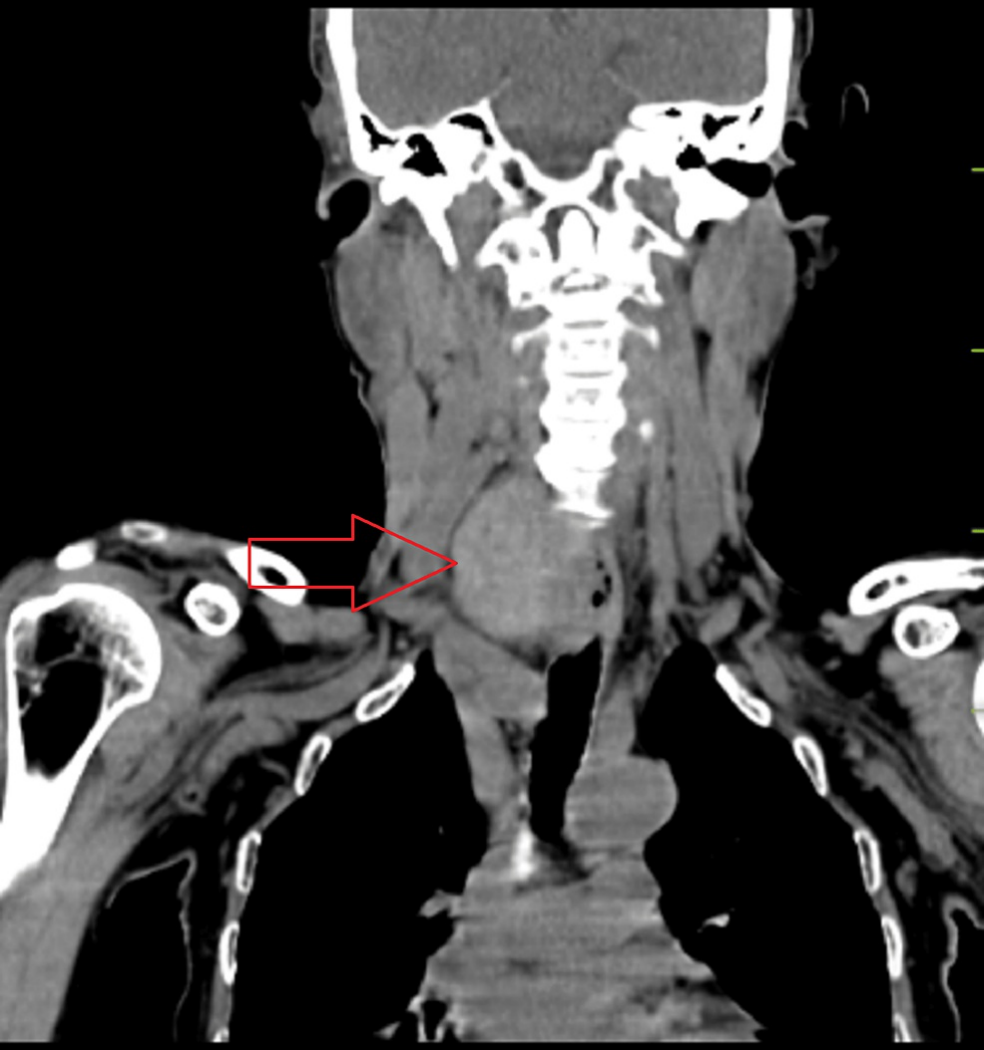
Contrast-enhanced computed tomography of neck Red arrow: Indicates iso dense swelling

On investigation, USG revealed that the left lobe of the thyroid is 12 x 9 mm and showed a well-defined iso to hyperechoic solid nodule with cystic changes measuring 6.6 x 6.6 mm with increased peripheral vascularity sign of multinodular goiter. On the USG neck, the right lobe of the thyroid appeared enlarged and measured 56*33mm and showed multiple iso to hyperechoic nodules with cystic changes of varying size, the longest of size 26*14mm with increased internal and peripheral vascularity. The isthmus of the thyroid was 1.2mm in size. On blood investigations, T3 was found to be increased, and TSH was normal. On HRCT thorax, a large-size heterogenous mass was noted in the right lobe of the thyroid extending up to T2 vertebral level measuring approximately 4.6 x 4.7 x 4.0 cm with a peripheral calcification group causing displacement and narrowing of trachea and esophagus toward the left side. There was a small heterogeneously enhancing nodule measuring 7.6 x 6.7 mm in the left lobe of the thyroid. The visualized skeleton shows degenerative changes, with compression fractures at T6, T8, and T12 vertebral levels. USG-guided fine-needle aspiration cytology revealed that cytomorphology suggests a nodule of adenomatous goiter. Right vocal palsy was noted on endoscopic examination during ear, nose, and throat assessment. X-ray of the neck with the arrow depicting the goiter is shown in Figure 1. CECT of the neck is illustrated in Figure 2.

Intervention

*Post-operative Goals* 

The short-term goal was to reduce the pain and discomfort, improve the lung patient's lung capacity and the cervical range of motion, and reduce the hoarseness of voice. The long-term goal is to improve the voice patient's voice-related quality of life.

Week 1

Basic bed mobility exercises were taught to the patient. Simple active movements of the upper and lower limbs were trained to enhance and sustain blood circulation. Breathing activities, including thoracic expansion and deep breathing, were taught to prevent pulmonary complications. For an additional week, lower limb mobility activities were continued to avoid secondary problems such as bed sores and deep vein thrombosis. These exercises included ankle-toe movements, static hamstrings, static quadriceps, and heel slides. Active cervical movements were encouraged. Isometric exercises for neck muscles were also started. Passive movements to the cervical spine in a pain-free range were also provided.

Week 2

Voice therapy exercises consist of deep inspiration and expiration exercises along with production of some sounds [13]. The exercises were given in two-step procedures. The description is as follows: 1. Voice exercise with relaxed breathing: Sit with your back straight and your neck and shoulders at a comfortable level. Gently inhale through your nose. To exhale, stick your tongue out of your mouth past the teeth and lower lip. The tongue's forward stretch aids the airway at the vocal cords. With a strong spasm, this could be challenging, but with practice, it will get easier. Exhale solely through the mouth in slow, stopped, or spaced breaths while keeping the tongue out. The rhythm should be like gently saying, "Ha, Ha, Ha, Ha." Just let your air out without speaking. Practice three times daily and say it 10 times. 2. Voice exercise with diaphragmatic breathing: Perform the diaphragmatic breathing exercise as you exhale after the deep inspiration exercise. Put the end of the tongue where the upper teeth meet the roof of the mouth. This will enable you to exhale with a hissing or "S" sound. Maintaining the airway's openness causes back pressure. Push the air between your tongue and teeth to produce a hissing or "S" sound as you slowly exhale while allowing the hand and belly to glide inward to a resting posture. Practice three times daily and repeat 10times.

*Week 3-4* 

In the third and fourth weeks, the exercises prescribed in week one and week two were continued, and the progression of strengthening of cervical muscles was continued; shoulder shrugs, neck isometrics, and shoulder-scapular exercises were all taught as ways to avoid compensatory posture. Repetition of consonant and vowel pairing exercises was added to the protocol. It consisted of a difficult-to-pronounce consonant and then matching it with each of the five vowels (a, e, i, o, u) and, for instance, repeatedly saying "ra, re, ri, ro, ru" if there was a problem with the "r" sound.

Follow-up and outcome

The patient could perform all her daily activities without any discomfort or complications. The patient's health significantly improved after four weeks of integrative physiotherapy strategies. There was a significant reduction in pain on the NPRS scale, increased range of motion to near-normal state, and improved voice-related quality of life questionnaire. The comparison of the pre-treatment and post-treatment scores is shown in Table 3.

**Table 3:** Outcome measures table

1.Numerical pain rating scale	7/10	2/10
2.Range of Motion: 1. Cervical Flexion	60°	80°
2. Cervical extension	50°	70°
3. Rotation: Right	70°	90°
Left	70°	90°
4.Lateral flexion: Right	35°	45°
Left	35°	45°
3.Voice-related quality of life	25/100	50/100

## Discussion

This case report describes physiotherapy rehabilitation after a total thyroidectomy of a 65-year-old female who suffered from multinodular goiter. Post-operatively, she had unilateral vocal cord paralysis. Unilateral vocal cord paralysis is one of the leading complications following a thyroidectomy procedure [14]. Injury to the recurrent laryngeal nerve causes vocal cord paralysis. The recurrent laryngeal nerve is known to carry motor impulses to the vocal folds. Damage to this nerve causes the failure of motor impulses to be carried from the vocal cord hence leading to vocal cord palsy. Hoarse voice, difficulty with high pitch, cough, aspiration, or a mix of these symptoms are how patients typically present when they suffer from vocal cord paralysis [15]. Vocal cord paralysis lowers the patient's quality of life. It was found that with early administration of voice therapy, vocal function and glottal closure were enhanced [16]. Voice therapy sessions benefit individuals with superior laryngeal nerve damage and those with unilateral or bilateral recurrent laryngeal nerve palsies with sufficient or minimal glottal competence. It helps them enhance voice production [17]. In a case study of tumor-induced compression of the laryngeal nerve, it was found that rehabilitation combined with vocal therapy significantly improved the patient’s condition from complete difficulty in producing sound to normal conversation [18]. Vocal and physical therapy exercises are noninvasive and a conservative line of management. Physiotherapy exercises combined with voice therapy exercises are a new territory which needs to be explored. Rehabilitation of a patient suffering from unilateral vocal cord palsy using physical therapy techniques is an out-of-the-ordinary idea. This case report might open the door to unexplored areas from the physiotherapy point of view. This case report proves that voice therapy exercises combined with physiotherapy improve the patient’s voice-related quality of life.

## Conclusions

The patient improved significantly following a rehabilitation program combined with vocal therapy exercises. Her voice-related quality of life questionnaire as well as a cervical range of motion demonstrated a significant improvement. Successful rehabilitation using vocal therapy exercises was done. Vocal exercises combined with relaxed breathing, diaphragmatic breathing, and consonant and vowel repetition exercises should be considered for treating a patient with vocal disorders.
